# Computational analysis of stochastic heterogeneity in PCR amplification efficiency revealed by single molecule barcoding

**DOI:** 10.1038/srep14629

**Published:** 2015-10-13

**Authors:** Katharine Best, Theres Oakes, James M. Heather, John Shawe-Taylor, Benny Chain

**Affiliations:** 1 Division of Infection and Immunity, UCL, London; 2 CoMPLEX, UCL, London; 3 Department of Computer Science, UCL, London.

## Abstract

The polymerase chain reaction (PCR) is one of the most widely used techniques in molecular biology. In combination with High Throughput Sequencing (HTS), PCR is widely used to quantify transcript abundance for RNA-seq, and in the context of analysis of T and B cell receptor repertoires. In this study, we combine DNA barcoding with HTS to quantify PCR output from individual target molecules. We develop computational tools that simulate both the PCR branching process itself, and the subsequent subsampling which typically occurs during HTS sequencing. We explore the influence of different types of heterogeneity on sequencing output, and compare them to experimental results where the efficiency of amplification is measured by barcodes uniquely identifying each molecule of starting template. Our results demonstrate that the PCR process introduces substantial amplification heterogeneity, independent of primer sequence and bulk experimental conditions. This heterogeneity can be attributed both to inherited differences between different template DNA molecules, and the inherent stochasticity of the PCR process. The results demonstrate that PCR heterogeneity arises even when reaction and substrate conditions are kept as constant as possible, and therefore single molecule barcoding is essential in order to derive reproducible quantitative results from any protocol combining PCR with HTS.

The efficiency of a PCR reaction is known to vary widely, depending on many different factors. These include the properties of the primers^1^,^2^,^3^, the sequence to be amplified^4^, in particular the GC content^5^,^6^, as well as the reaction conditions and type of polymerase. If one wishes to quantify the amount of a given template by PCR (qPCR) the general approach is to compare an unknown sample to a dilution series of standards, on the assumption that all variables remain the same between sample and standard and hence PCR efficiency remains constant.

The introduction of high throughput sequencing (HTS)^7^,^8^, in which many DNA molecules are sequenced individually in parallel, allows the possibility of quantifying many initial target molecules simultaneously by counting the number of times the sequence for each molecule occurs in a sequence run. This approach forms the basis for RNA-seq, in which transcript abundance is measured by sequencing cDNA libraries, and counting the number of sequences mapping to each transcript. An extension of this approach is the analysis of the antigen-specific receptor repertoire by sequencing cDNA or genomic samples of B or T lymphocytes, and counting the number of times each different receptor is identified^9^. Most current parallel sequencing technologies require nanomolar amounts of starting material (typically >10^10^ molecules), even when the output of the reaction may only be in the order of 10^7^ molecules (for example the Illumina MiSeq). In order to achieve this amount of starting material some degree of PCR amplification is usually required. This is especially true when the amount of starting material may be extremely small, for example in the case of single cell RNA-seq^10^. The reproducibility of the PCR amplification process therefore becomes a key factor for accurate quantification.

The use of molecular barcodes provides one approach to dealing with single molecule quantification and mitigating the effects of PCR heterogeneity. A library of diverse short DNA sequences (barcodes or tags) are introduced into the molecules to be analysed at an early step in the protocol, in such a way that each target molecule incorporates a different tag which remains associated with it throughout the amplification protocol. The barcodes can be introduced during a reverse transcription step, or by ligation. For instance, Miner *et al.*^11^ and McCloskey *et al.*^12^ both ligate nucleotide sequences to uniquely label initial DNA target molecules to identify sequencing redundancy as well as using batch stamps to identify sequencing contamination from other samples. In the work of Casbon *et al.*^13^ degenerate base regions are ligated to each DNA fragment to assess whether observed differences between sequence reads are true variants or sequence error. Kivioja *et al.*^14^ apply a similar unique molecular identifier technique to human karyotyping. Mamedov *et al.*^15^ and Shugay *et al.*^16^ use barcodes to provide PCR and sequencing error correction of TCR repertoires.

In this study we use molecular barcoding to investigate the extent of variation in PCR amplification on a single molecule basis. In order to rigorously assess the possible sources of this heterogeneity we develop an efficient PCR simulator, which incorporates both amplification and sampling heterogeneity, with which to compare our experimental results. The PCR amplification is an example of a branching process, and although there has been considerable theoretical work on such processes, the complexity of heterogeneous branching processes makes analytical modelling challenging in most realistic examples^17^,^18^,^19^. Detailed models of the physical parameters involved in the PCR cycles have been developed, to answer questions about the probability of replication in an individual cycle or the evolution of the population over a number of cycles^20^,^21^,^22^,^23^,^24^. Additionally mathematical models have been used to investigate the error profile in PCR protocols^25^ or the presence of non-targeted product through non-specific priming^26^. An increase in computing power has made it feasible to develop PCR simulations using realistic numbers of starting molecules, with reasonable run times. The model we describe includes both an amplification step and a sampling step to simulate the typical workflow of an RNA-seq or repertoire sequencing experiment. These computational tools can distinguish heterogeneity which derives simply from the sampling process itself (modelled as a zero truncated Poisson process) from stochastic variation in each step of the PCR reaction and inherited variation which may arise from differences between different DNA molecules within the reaction. Our study therefore highlights the potential pitfalls in quantitative analysis of DNA or RNA abundance involving a PCR amplification step, and provides a computational framework which can be used to analyse barcoded PCR data, and identify and quantify the sources of heterogeneity.

## Methods

### Ethics

This study was approved by the joint UCL/University College London Hospitals NHS Trust Human Research Ethics Committee and was carried out in accordance with relevant guidelines and regulations. Written informed consent was obtained from all participants (University College Hospital 06/Q0502/92).

### Sample collection

5 ml of healthy adult volunteer blood was drawn into Tempus Blood RNA tubes (Life Technologies) and RNA was extracted using the Tempus RNA isolation kit (Life Technologies). Residual DNA was removed using the TURBO DNase kit, and globin mRNA was depleted using GLOBINclear (both Life Technologies).

The KT2 T cell clone was a gift of Prof. A. Lanzavecchia (Institute for Research in Biomedicine, Bellinzona, Switzerland). The clone was grown as described^27^. RNA was isolated using the RNeasy Mini Kit (Qiagen). RNA was treated with RQ1 DNase (Promega) following manufacturer’s instructions to remove any residual genomic DNA.

Two different protocols were used to amplify and then sequence the T cell receptor chains. All primers are from Sigma-Aldrich and sequences can be found in Table 1.

### Protocol using single strand ligation (Protocol A)

The DNAse treated RNA was reverse transcribed using oligos complimentary to the 5′ region of the TCR constant regions TRAC and TRBC (αRC2 and βRC2). The mastermix for the reverse transcription was added to the RNA in two stages (molarities for both mastermixes relate to the final volume of 30\mu l). 11 μl of DNase treated RNA were mixed with 0.5 μM αRC2, 0.5 μM βRC2 and 0.5 mM of each dNTP (Invitrogen) to total 19.5 μl, and then incubated at 65 °C for 5 min and cooled rapidly on ice for >1 min. 1× FS buffer (Invitrogen), 5 mM DTT (Invitrogen), 30–60 units RNasin Ribonuclease Inhibitor (Promega) and 300 units SuperScript III reverse transcriptase (Life Technologies) were added before incubation at 55 °C for 30 min in a total volume of 30 μl. 40 mM NaOH were added to remove any remaining RNA and the sample was incubated at 70 °C for 15 min. 0.5 M sodium acetate were added to adjust the pH before the cDNA reverse transcription product was purified using MinElute columns (Qiagen).

The single stranded cDNA was ligated, using T4 RNA ligase (NEB) to a 5′ phosphorylated 3′ blocked oligonucleotide (T4DNA_6N_SP2 in Table 1) containing 6 base pairs of random nucleotide barcode and the Illumina sequencing primer SP2. 5 μl of cDNA were mixed with 1x T4 RNA ligase buffer (NEB), 1mM hexammine cobalt chloride, 1.5 μM BSA, 0.33mM ATP (NEB), 0.33 μM ligation oligo and 20 units T4 RNA ligase 1 (NEB). The ligation was carried out at 16° C for 23 hours followed by a 10 minute heat inactivation step at 65° C. 70 μ l water were added to the ligation mix before samples were purified at a 1:1 ratio with AMPure XP SPRI beads (BeckmanCoulter) following manufacturer’s instructions and eluted in 30–35 μl water. A second strand was then synthesised, priming from the ligated SP2 sequence. The AMPure bead purified ligation product was incubated with 1x HF buffer, 0.5 μM SP2 primer, 0.5 mM of dNTPs and 1 unit of Phusion polymerase in a 50 μl reaction at 98 °C for 3 min, lowered slowly (1 °C/sec) to 80 °C, held at 80 °C for 10 sec, lowered slowly (1 °C/sec) to 58 °C and held at 58 °C for 30 sec. After the final extension at 72 °C for 1 min, the product was again purified on AMPure beads.

**Figure 1:**
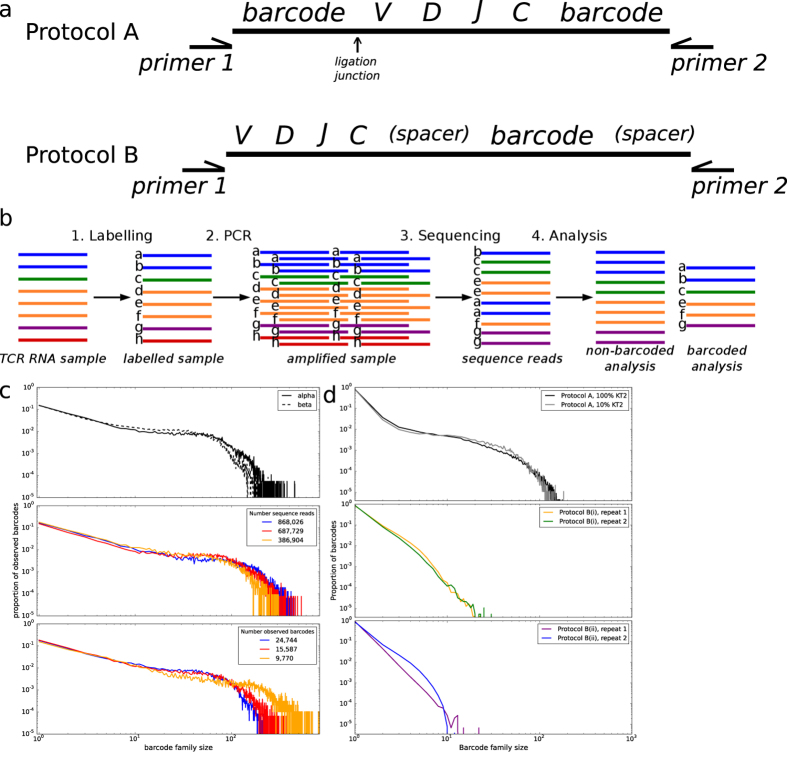
(**a**) Schematic of the target TCR molecule in Protocols A and B showing the position of the barcode for molecular identification and the PCR priming sites. In the T cell receptor portion of the molecule, V, D, J and C refer to the Variable, Diversity (β chain only), Joining and Constant regions (not to scale). The two alternative possible positions for the barcodes in protocol B(i) and B(ii) are shown in brackets. The Illumina sequencing primers, indices to allow for multiplexing of samples, and the Illumina adaptor sequences are not shown. (**b**) Schematic of experimental and computational protocol used to sequence and analyse TCRs from isolated RNA. Barcodes (represented here by lower case letters) are included in each TCR molecule together with a known sequence (SP2). PCR is then performed to amplify the sample. The amplified pool of molecules is diluted and introduced to the sequencer, where a sample of molecules will adhere to the flow cell and be sequenced. Repertoire analysis is performed on the sequencing data, with the barcodes allowing correction of biased PCR amplification as well as correction of sequencing errors. (**c**) The distribution of observed barcode family size (the number of reads occurring in the sequencer output that originate from the same initial target molecule in the sample) in polyclonal TCR sequence data (Protocol A) from healthy volunteer T cells. Upper: TCR alpha chain (solid line) and beta chain (dotted line) data. Middle: TCR repertoires sequenced at different depths Bottom: TCR repertoires with different numbers of observed barcodes, representing the number of initial molecules. (**d**) The observed barcode family size distribution observed in TCR sequence data from a sample of RNA isolated from a T cell clone (KT2, responding to tetanus toxoid^27^). Upper: TCR alpha chain (solid line) and beta chain (dashed line) from protocol A. Middle: TCR beta chain data from protocol B, using the oligonucleotide with 6 bp spacer between the sequencing primer and the barcode. Bottom: TCR beta chain data from protocol B, using the oligonucleotide with the barcode directly next to the sequencing primer.

An additional random six base pair barcode was added to the 3′ end with a third strand synthesis. The conditions for third strand synthesis were identical to second strand, but an using an oligonucleotide complimentary to the constant region, and an extension containing the random barcode, and the Illumina SP1 sequencing primer (SP1-6N-I-X-αRC1, or SP1-6N-I-X-βRC1). A diagram showing the structure of the DNA at this point is shown in Fig. 1a (top).

The barcoded TCR samples were then amplified in two different consecutive PCR reactions. In the first PCR the P5 and P7 adapters required for Illumina sequencing, and an index for multiplex sequencing were added with primers P5-SP1 and P7-LX. The PCR conditions used were 1x HF buffer, 0.5 μM P5-SP1, 0.5 μM P7-LX, 0.5 mM dNTPs and 1 unit Phusion; initial cycle: 98 °C for 3 min, slowly ramped to 69 °C for 15 sec and 1 min at 72°; cycle 2–4: 98 °C for 10 sec and 72 °C for 1 min; final cycle: 72 °C for 5 min. After bead purification the samples were amplified in a second PCR (1x HF buffer, 0.5 μM P5s (details below), 0.5 μM P7 (details below), 0.5 mM dNTPs and 1 unit of Phusion); initial cycle 98 °C for 3 min; cycle 1–24: 98 °C for 10 sec, 69 °C for 15 sec, 72 °C for 40 sec; final cycle: 72 °C for 5 min. PCR2 products were bead purified and eluted in 30 μl water.

Protocol A is represented schematically in Supplementary Fig. 5.

### Protocol using fixed V region primer (Protocol B)

DNAse treated RNA isolated from the KT2 clone was reverse transcribed using oligos complementary to the 5′ region of the TCR β constant region TRBC. The oligonucleotides also contained a random 12 base pair barcode, the SP1 Illumina sequencing primer and an index for multiplexing. We used two different indices, and each index was placed either next to the SP1 primer sequence (thus providing a spacer between primer and random barcode; SP1-12N-IX-βRC1.1; Protocol B(i)), or adjacent to the constant region sequence (SP1-IX-12N-βRC1.1; Protocol B(ii)). Reverse transcription was carried out as in Protocol A.

The cDNA was amplified using a KT2 V region specific primer (VBKT2_1, Table 1) and an oligonucleotide complimentary to the Illumina Sequencing Primer SP1. PCR conditions were 1x HF buffer, 2.5 μM primers, 0.5 mM dNTPs and 1 unity Phusion; initial cycle 98 °C for 3 min; cycle 1–24: 98 °C for 10 sec, 69 °C for 15 sec, 72 °C for 40 sec; final cycle: 72 °C for 5 min. PCR products were bead purified and eluted in 30 μl water. The P5, P7 and multiplex index elements were added in 4 additional rounds of PCR as described above.

### Library sequencing

Final amplicon products from all sample types were quantified on a Qubit fluorometer (Life Technologies) and sized on a Bioanalyzer (Agilent). Up to 12 samples (at a concentration of 4 nM) were multiplexed and sequenced on an Illumina MiSeq, using version 2 chemistry 2x 250PE kits.

### Data analysis

The FASTQ files produced on the MiSeq were demultiplexed based on the indices added through PCR and analysed using a modified version of Decombinator^28^. Decombinator categorises each TCR sequence read by identifying its constituent V gene and J gene, along with the number of nucleotide deletions from each and the non-germline junctional nucleotides inserted during TCR recombination. The five-part Decombinator classifier (DCR) is given by: V gene used, J gene used, number of V deletions, number of J deletions, nucleotides between V and J. The modified version of Decombinator used in this study outputs the DCR along with information about the random nucleotide barcode and sequence quality in each sequence read.

For analysis of polyclonal TCR sequence data, the Decombinator output is then passed into a PCR- and sequencing-error correction script. This script first filters sequence reads to remove those where the barcode or sequence quality are poor. It then collects all sequence reads according to their barcode, grouping together those DCRs that appear with identical barcodes. If more than one distinct DCR appears with the same barcode, we take the DCR with the most copies to be the true sequence with that barcode, and the others are aggregated into the largest DCR if they are clearly the product of sequencing error or discarded otherwise. Next, the set of different barcodes associated with the same DCR is considered. Barcodes that are similar and are observed in the context of the same DCR are considered to be derived from the same initial molecule and are therefore aggregated. The size of the set of distinct barcodes found in the context of the same DCR provides us with a measure of the number of initial copies of that T cell receptor present in our sample (the clone size). For this study, we additionally count the number of copies of each barcode-DCR combination (the barcode family size) to provide us with information about the amplification of the initial molecules.

The structure of the available barcode pool is inferred from the distribution of the number of times each barcode is found to have labelled a different cDNA molecule (barcode-labelling events) across all experiments in this study. The barcode-labelling events data are fitted by various zero-truncated mixed Poisson models using custom functions (found in Supplementary Information), minimised using the Optimise function of SciPy in Python. The parameters of the fitted models are used to infer the structure of the pool of available barcodes.

### PCR simulator

Simulation of labelling, amplification and sequencing of samples of molecules is performed with functions written in Python and available at github.com/uclinfectionimmunity/PCRsim. Briefly, at each cycle a molecule has a chance to successfully replicate. The probability of successful replication is determined by the PCR model chosen. If replication is successful, nucleotide error is incorporated at a given rate by choosing at random whether a given position in the sequence contains error and if so which nucleotide is incorporated incorrectly. Molecules to be sequenced are selected at random from the amplified pool and sequencing error is incorporated into these molecules similarly.

## Results

### Heterogeneous amplification efficiency demonstrated by unique molecular barcoding of cDNA molecules

We reverse transcribed a sample of TCR RNA from peripheral blood T cells and then ligated a primer that contained a unique barcode followed by a sequence corresponding to the Illumina SP2 sequencing primer (Protocol A). The individually tagged mixtures of different α and β chains were amplified using constant region 3′ primers and a 5′ primer homologous to the Illumina SP2 sequence on the ligated oligonucleotide (Fig. 1a top). The resulting amplified PCR reaction was diluted and sequenced using the standard Illumina protocol (illustrated diagrammatically in Fig. 1b). The number of times each barcode was present in the sequence data was then counted. We refer to all sequences that have an identical barcode as a barcode family, and refer to the number of molecules present with this barcode as a barcode family size. Although each cDNA molecule was ligated to a different barcode, and the starting frequency of each barcode should then be uniform and independent of the frequency of the TCR sequence with which it was associated, the observed distribution of barcode family sizes in a polyclonal sample was very heterogeneous (Fig. 1c, top). Thus, while the majority of barcode families were of size one, some barcodes occurred over 100 times. A similar pattern was observed for α and β TCR sequences, indicating that the heterogeneity was not some special feature of the sequence being amplified. We repeated this analysis on different polyclonal samples, sequenced at different depths (Fig. 1c, middle) and with different numbers of observed barcodes (and therefore different numbers of initial target molecule) carried through the protocol (Fig. 1c, bottom). Extensive heterogeneity, varying over two orders of magnitude, was observed in each case. Without barcoding, this heterogeneity would have a substantial impact on analysis of both the diversity and the structure of the TCR repertoire (Supplementary Fig. 2).

One explanation for the observed distribution was the heterogeneous template mixture of cDNAs due to the diversity of the TCR repertoire. Although the primers and the primer binding regions were the same for all amplified molecules, the intervening sequences were heterogeneous since they represented many different TCR sequences. Thus, heterogeneous amplification could reflect differences in target replication by polymerase. In order to simplify the experimental model, and limit the heterogeneity arising from using a complex pool of substrate molecules (a natural TCR repertoire), we labelled and amplified a TCR sequence (α and β chain) from a human T cell clone, KT2, which expresses only one T cell receptor. As predicted, the vast majority of sequences from these samples were identical. (Supplementary Fig. 1). To our surprise the distribution of barcode frequencies was still just as heterogeneous (Fig. 1d, top). Thus even under conditions where we were amplifying a single target (namely the KT2 TCR α or β chain), and primer and reaction conditions were identical for all amplified molecules, we still observed a difference of two orders of magnitude in the number of molecules derived from single starting template cDNA molecules.

We considered two further sources which could potentially contribute to the observed heterogeneity in amplification efficiencies. The first was the single-stranded DNA ligation step used in Protocol A (Fig. 1a, top). Although this allows a single primer to be used for a heterogeneous mixture of DNAs and avoids the need for complex primer multiplexing, it creates a potential for heterogeneity at the end of the cDNA template molecule as a result of incomplete reverse transcription of the RNA. A second possible cause of heterogeneity are the barcodes themselves. In particular Pan *et al.*^3^ have shown that the basepairs immediately adjacent to the PCR primer can have a small effect on amplification efficiency.

In order to address the first issue, we performed a further PCR using a fixed primer within the V region of the KT2β chain instead of the single stranded ligation step (Protocol B). The unique barcodes were introduced during the RT step, and were placed either adjacent to the primer as previously, or separated from the primer by a six base pair index region (Fig. 1a, bottom panel). The results of these further sets of PCR are shown in Fig. 1d (middle and bottom panels). The omission of the ligation step decreased the amount of heterogeneity, although differences in amplification of greater than 10 fold remained.

### Barcode family size is not dependent on barcode sequence, barcode clash or non-uniform barcode primer frequencies

**Figure 2:**
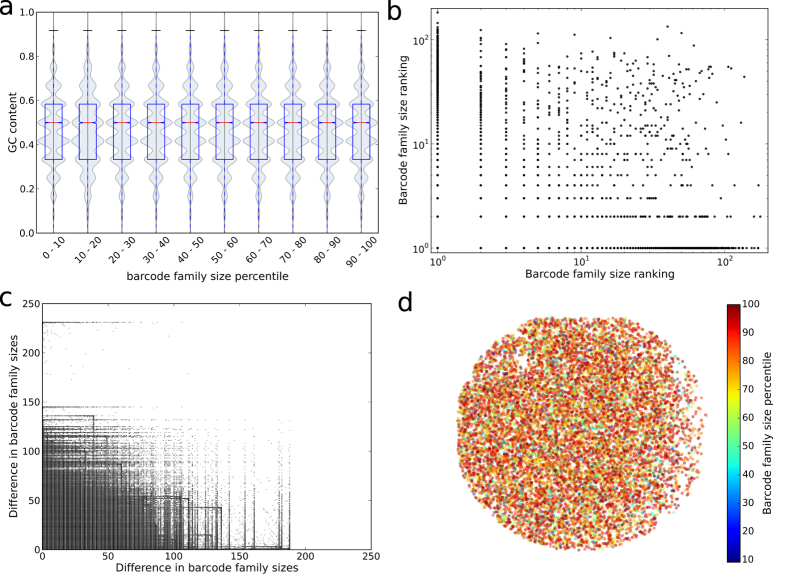
(**a**) Distribution of GC content of the 12-nucleotide random barcodes by barcode family size percentile. Data from a sequence run of healthy volunteer PBMC TCRs. (**b**)The correlation between the barcode family size ranking in any pair of runs for those barcodes that occur in more than one of the eight monoclonal KT2 TCR sequencing runs (Protocol A) in this study (R-squared <0.0003). Ranking is ascending, and barcodes that have the same family size in a run are given the same ranking. There is no gap introduced in rankings when more than one barcode occupies a particular ranking, as such for small barcode family sizes ranking is equivalent to barcode family size. (**c**) For those pairs of barcodes that appear together in any pair of the eight KT2 sequencing runs (Protocol A) in this study, the relationship between the difference in barcode family sizes in one run and in the other. R-squared <0.0004. (**d**) Position of TCR molecules on the flowcell, coloured by barcode family size percentile. Representative example of a single frame from one flow cell from a sequencing run in this study.

The heterogeneous amplification observed could hypothetically be caused by the barcode itself since the polymerase must amplify the barcode in each cycle. To investigate this, we first considered whether barcodes that appear more amplified have a tendency to contain more or fewer G or C nucleotides (Fig. 2a). However, there was no obvious relationship between the frequency of particular barcodes and their GC content. Furthermore, the frequency of the same barcode in any two different sequence runs was uncorrelated (Fig. 2b). A high barcode family size did not therefore appear to be the result of a particular barcode sequence or sequence motif. Additionally, to account for the fact that the amplification effect might be to do with relative, rather than absolute, barcode ‘fitness’, we considered all pairs of barcodes that both appear in any pair of experiments. If the amplification was determined by the barcode we would expect, for example, that if barcode A is larger than barcode B in experiment 1 then it would also be larger in experiment 2. We found no correlation between the frequencies of any two barcodes that appear together in a pair of experiments (Fig. 2c), implying that the barcode sequence itself does not determine the efficiency with which each molecule is amplified. We also examined whether the observed barcode family size might be an artefact introduced during the sequencing reactions, perhaps by heterogeneity in bridge PCR on the flow cell. If this were the case we would expect that molecules from large barcode families are located in close proximity on the flow cell. However there was no observable relationship between barcode family size and location of molecules on the flow cell (one representative frame shown in Fig. 2d).

**Figure 3:**
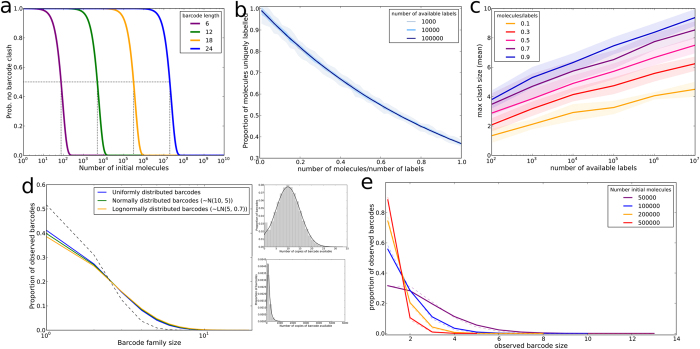
(**a**)Probability that no two molecules receive the same barcode (“barcode clash”) when labelled with random nucleotide barcodes of the indicated length. Dotted lines: number of molecules that can be labelled with a 50% chance of no barcode clash. (**b**) Proportion of molecules that receive a unique barcode when labelling is simulated with the indicated number of available barcodes, uniformly distributed. Number of molecules to be barcoded is expressed as a proportion of the number of available barcodes. Data shown is mean and standard deviation of 50 repeated simulations. (**c**) Maximum number of initial molecules that receive the same barcode when barcoding is simulated with the indicated number of available barcodes, uniformly distributed. Number of molecules being barcoded is indicated by colour, expressed as a proportion of the number of available barcodes. Data shown is mean and standard deviation of 50 repeated simulations. (**d**) Observed barcode size distribution after simulation of labelling 250,000 molecules from uniformly or non-uniformly distributed pools of 500,000 available barcodes, 10 cycles of PCR (efficiency 0.5) and sampling 300,000 molecules from the amplified pool. Inset: distribution of available barcodes for non-uniform simulations (green: normal (restricted to values >0), orange: lognormal). Data shown are mean and standard deviation of 10 repeated simulations. Grey dotted line: expected distribution if the sampled molecules were drawn from a uniformly distributed amplified pool, in which all molecules had been uniquely barcoded and amplified equally. (**e**) Observed barcode size distribution when the indicated numbers of initial molecules are barcoded from a pool of 4^12^ potential barcodes with barcode availability distributed as predicted from empirical labelling events observed (Supplementary Information and Supplementary Fig. 3). 25 PCR cycles (efficiency 0.75) are simulated on labelled molecules and samples of 100,000 are selected from the amplified pool. Solid line: mean of 10 repeated simulations. Dashed line: expected distribution if the sampled molecules were drawn from a uniformly distributed amplified pool, in which all molecules had been uniquely barcoded and amplified equally.

The barcodes should theoretically contain randomly chosen nucleotides at each of the 12 positions, giving a total of 4^12^ ≈ 1.7 × 10^7^ possible barcodes, each appearing an equal number of times. In practice, the methods of oligonucleotide synthesis likely result in slightly different incorporation efficiencies of different nucleotides at each position^29^. In addition, the number of target molecules barcoded in our T cell samples is often within an order of magnitude of the number of available barcodes, resulting in a significant probability that the same barcode is used more than once (‘barcode clash’) (Fig. 3a). In order to assess the impact that this barcode clash might have on the observed barcode family sizes, we first simulated barcoding molecules from a large, uniformly distributed pool of available barcodes and measured the proportion of molecules that were uniquely barcoded (Fig. 3b). This value depends on the ratio of the number of available barcodes (size of the barcode pool) to the number of molecules to be barcoded. In these simulations we also measure the maximum observed barcode clash size (Fig. 3c), which in contrast also depends on the absolute number of available barcodes and molecules to be barcoded. These simulations show that in our protocol (barcoding in the order of 10^6^ molecules with 10^7^ available barcodes) around 90% of molecules get a unique barcode and the maximum clash size is predicted to be below 4. Thus barcode clash is unable to account for the range in barcode family sizes we observe in our data.

It is likely that the pool of barcodes we have available for labelling is not exactly uniformly distributed, which could lead to increased barcode clash. We simulated the barcoding, amplification and sequencing protocol using normally or lognormally distributed barcode frequency distributions, but this had little effect on the observed barcode family size distributions when compared to uniquely barcoding every molecule or to the expected distribution if every initial molecule was represented equally in the post-PCR amplified pool (Fig. 3d). We also derived the empirical distribution of barcodes in our initial oligonucleotide pool (see Supplementary Information and Supplementary Fig. 3) and simulations using this distribution do not show a barcode family size distribution deviating far from the sampling distribution expected from a uniformly distributed amplified pool (Fig. 3e). The output of the barcoding, amplification and sequencing pipeline is therefore robust to the likely occurrence of barcode clash and non-uniform barcode frequencies.

### Inherited differences in PCR efficiency are necessary to explain the observed diversity in barcode family size

**Figure 4:**
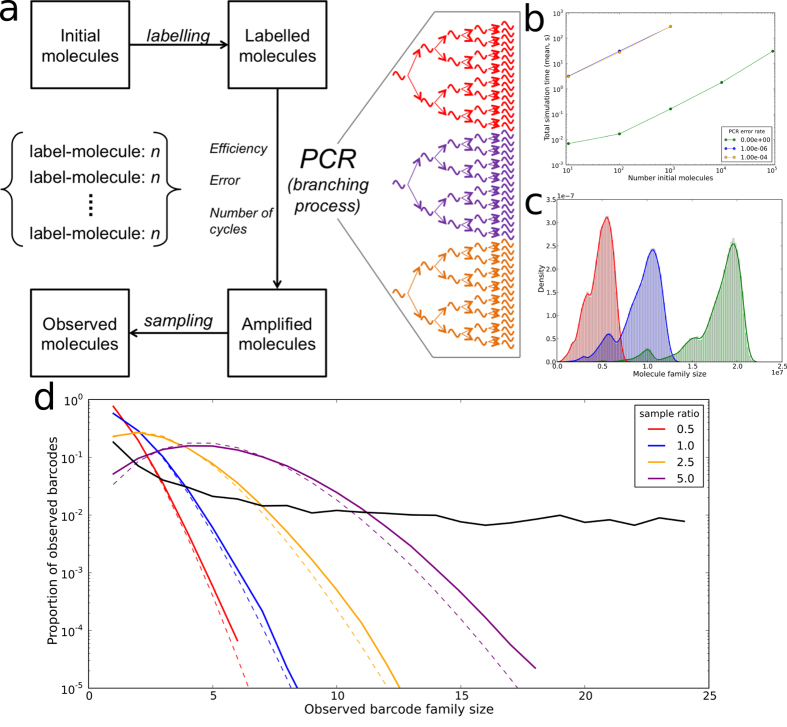
(**a**) Schematic of the PCR simulator software used in this study. The software includes adding barcodes to molecules (‘labelling’), PCR amplification with a specified number of cycles, efficiency model and error rate, and sampling and sequencing from the amplified pool. (**b**) Time taken to perform a full simulation, which includes initialisation, labelling initial molecules, PCR cycles (using a standard branching process model), sampling from the amplified pool and sequencing. Simulations are performed with the indicated PCR error rate (per base per cycle) and the given number of initial template molecules. Simulations consist of 15 cycles of PCR with efficiency 0.8, a sample size equal to the number of initial molecules being chosen from the amplified pool and sequencing with error rate 10^−4^. Data shown is the mean of 5 repeated simulations at each set of conditions, as measured on a 2.8 GHz Intel Core i7 MacBook Pro. (**c**) The distribution of the number of copies of each of 100,000 initial target molecules after 25 cycles of PCR at efficiencies of 0.85 (red), 0.9 (blue) or 0.95 (green). (**d**) The distribution of observed barcode family sizes (coloured lines) after simulating PCR cycles (25 cycles at 0.9 efficiency) on 100,000 initial molecules and then sampling from the amplified pool to select those molecules that are observed in the sequencer output. The number of molecules sequenced is expressed as a proportion (the ‘sample ratio’) of the number of initial molecules (100,000). The solid coloured lines are the mean of 5 repeated simulations, and the dashed coloured lines are the expected distribution (a zero truncated Poisson with parameter equal to the sample ratio) if the sample was drawn from a uniformly distributed pool (which would occur if every initial molecule was uniquely barcoded and amplified identically). The black solid line is a representative example of the barcode family size distribution observed in TCR sequencing data from healthy volunteer PBMC.

The experimental pipeline involves amplification followed by subsampling for sequencing. There are two distinct sources of stochasticity in the pipeline. The subsampling of sequences from the PCR product for sequencing introduces one source of heterogeneity. In addition, PCR efficiencies of less than 100% can introduce non-uniformity resulting from the inherent stochasticity of the PCR process^30^. In order to examine how each of these two sources of heterogeneity, namely variable efficiency and sampling could affect observed barcode family size distributions we developed a PCR simulator in which molecules are barcoded, amplified and then sampled in silico. The simulator is outlined schematically in Fig. 4a. In its most basic implementation (modelling PCR as a straightforward branching process with no error) the simulator can perform a full simulation (labelling initial molecules, performing 15 PCR cycles with efficiency 0.8, sampling and sequencing including sequencing error) on 10^5^ initial molecules in approximately 12 seconds on a standard specification laptop (Fig. 4b). Introducing PCR error substantially increases the simulation time, although altering the error rate further does not alter simulation time. Parallelisation and cluster research computing platforms make PCR simulation including error of large numbers of initial molecules feasible.

The simulated barcode distributions (the number of molecules present after amplification that are derived from each initial molecule) at different efficiencies are shown in Fig. 4c. The introduction of less than 100% efficiency introduces some barcode family size heterogeneity as described previously^30^. This variation arises because, in every replication cycle, any individual molecule may or may not replicate with a probability determined by the overall efficiency. The substantial shoulder observed in the distributions correspond to molecules which fail to be replicated in the first cycle of PCR and hence are present at half the average number of copies. However, the heterogeneity caused by low efficiencies is averaged out over many molecules and the majority of barcode family sizes are within a factor of two of each other at the end of the PCR reaction.

We next examined the influence of subsampling. In order to sequence the PCR product, molecules from the amplified sample are diluted and introduced to the flow cell to anneal to complementary capture oligonucleotides. This introduces heterogeneity into barcode numbers, which can be modelled by a Poisson distribution (as an approximation to a binomial distribution), scaled to account for the fact that we cannot count those barcodes with an observed family size of zero (a zero-truncated Poisson). In order to investigate the effects of sampling independently from those of PCR efficiency, we simulate samples taken from PCRs of 100% efficiency. The simulations were carried out at different ratios of number of sequenced molecules to initial molecules. The distributions obtained are compared in Fig. 4d to those observed from the Protocol A data. It is immediately obvious that our data from Protocol A does not belong to the same family as the simulated distributions (Fig. 4d). In order to obtain statistical support for this conclusion, we fitted the best Poisson distribution to each set of experimental data using maximum likelihood to select the Poisson parameters. A comparison of the optimum fitted Poisson to the data using chi squared test rejected the null hypothesis (p = 0). These results strongly suggest that these data were not Poisson distributed, and that the sampling process cannot account for the broad distribution observed.

**Figure 5:**
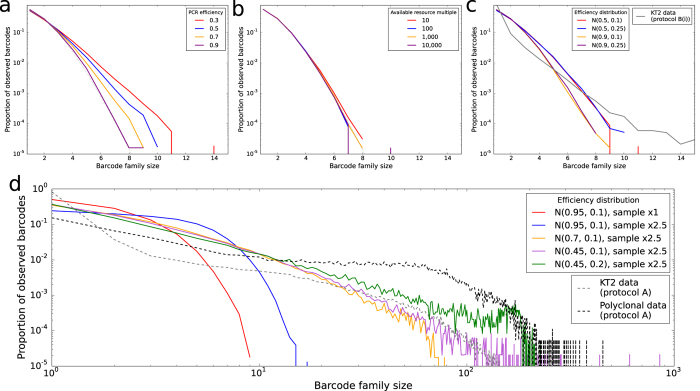
Observed barcode family size distributions observed under different models of PCR amplification. Simulations performed with 10,000 initial molecules, 25 cycles of PCR (with no error) and sequencing of 10,000 molecules selected from the amplified pool. Simulations were repeated 10 times and the mean and standard deviation are shown. The dotted lines represent the expected distribution if every initial molecule is barcoded uniquely and represented equally in the amplified pool. Grey and black lines represent the distributions observed experimentally in the indicated experiments. Models described in the text but not displayed here can be found in Supplementary Fig. 4. (**a**) Model 1: Standard branching process of PCR, with the indicated efficiencies. The efficiency is the probability that a given molecule will duplicate in a given cycle. (**b**) Model 3: Model of PCR where the duplication efficiency depends on competition between target molecules for a constant level of resource, given as a multiple of the number of initial molecules. (**c**) Model 5: Variable efficiency model of PCR, where the probability of a given molecule replicating in a given cycles is selected from a normal distribution (restricted to [0, 1]) with the indicated parameters (mean and standard deviation). (**d**) Model 6: Inherited efficiency model of PCR, where the probability of replication in a given cycle is identical for all molecules derived from the same initial molecule. The efficiencies for the initial molecules are selected from a normal distribution with the indicated parameters (mean and standard deviation). 25 PCR cycles are simulated on 10,000 initial molecules, and then a sample is drawn from the amplified pool at a multiple of 1 times or 2.5 times the number of initial molecules. The observed barcode family size distribution shown is the mean of 10 repeated simulations.

We therefore tried to formulate variations of the branching process model of PCR that could explain the broad barcode family size distribution observed. For each model, the distribution of barcode frequencies (Fig. 5) and the coefficient of variation of the barcode frequency (Supplementary Fig. 4) were compared between model and experimental data.

The starting point is a standard branching process model of PCR (‘model 1’) where the efficiency of the PCR (between 0 and 1) refers to the probability that a molecule will replicate successfully in a cycle. Using this model, we simulate PCR and sampling, and show that the resulting barcode family size distributions do not diverge significantly from the expected Poisson distribution regardless of the efficiency used (Fig. 5a). Next, a target degradation model (‘model 2’) was used. Model 2 is set up as for model 1, except that when a molecule fails to duplicate in a cycle there is a chance that it instead degrades and is no longer available to be amplified in later cycles of the PCR. Again, simulation of this model does not reproduce the large deviation from a Poisson distribution that is seen in our data (Supplementary Fig. 5a).

Next, we introduce competition for resource, which affects the success rate of duplication of molecules. This abstract ‘resource’ covers, for example, the availability of dNTPs and primer in the PCR mixture, and the ability of the enzyme to process the molecules inside the time frame given in the PCR protocol. The first resource competition model (‘Model 3’) is one in which there is a fixed, constant amount of resource available and the probability that a molecule successfully replicates in a cycle is given by the number of molecules present at the start of the cycle divided by the amount of resource. As such, the efficiency of the reaction decreases through the cycles once the number of molecules present exceeds the capacity of the available resource to process all those molecules in one cycle. Figure 5b shows that this model cannot reproduce the spread of barcode family sizes we observe in the data. An alternative resource competition model (‘Model 4’) involves degradation of resource as it is used, at a given degradation rate. This model is also unable to account for our observed barcode family size distributions (Supplementary Fig. 5b).

Instead of a constant efficiency across all molecules and all cycles, we imagine that in a given cycle some molecules are able to replicate more efficiently than others. For instance this variation may depend on the position of the molecule within the sample (which may affect e.g. proximity to primer) or the conformation of the molecule (which may affect ability of the primer to bind). We introduce a variable efficiency model (‘Model 5’), where the probability that a given molecule will replicate in a given cycle is chosen from a defined distribution. Model 5 is implemented using a normal distribution with a variety of parameters (Fig. 5c). A low mean efficiency and a large standard deviation produces the most divergence from the expected barcode family size distribution, and is able to account for the majority of the spread seen in barcode family size observed in the KT2 data from Protocol B. However, none of the parameters investigated was able to reproduce the observed spread of family sizes observed in polyclonal or monoclonal data from Protocol A.

We adapted Model 5 to include the constraint that once efficiency is chosen for a molecule in cycle 1 this same efficiency is inherited by all molecules produced from this initial molecule (‘Model 6’). Simulation of PCR and sampling using model 6 was performed, and showed that inherited efficiencies could produce a substantial amount of spread in the barcode family size distribution when the efficiency distribution has a low mean and a relatively large standard deviation (Fig. 5d). The observed barcode family size distribution from Model 6 can be seen to be broadly comparable to that seen in our experimental data (from Protocol A) for these figures. In contrast, the distribution which arises from model 5 is sufficient to account for most of the heterogeneity observed when using a fixed primer instead of ligating a primer to the end of the cDNA (Protocol B).

## Discussion

PCR is a fundamental and ubiquitous tool of molecular biology laboratories. The combination of PCR and HTS, in particular, has driven an explosion in DNA sequence acquisition. In many of these applications, for example RNA-seq and lymphocyte antigen receptor repertoire studies, the quantification of transcripts is critical, since the output is based on counts of specific sequences. The avoidance of PCR bias is therefore critical and much effort has been expended on trying to control and mitigate bias. In this study, we examine the consistency of PCR amplification, using molecular barcodes to follow amplification of single molecules. We find that the distribution of the number of copies of an initial molecule that are observed in sequencer output varies over a wide range, even when primers, target sequence, bulk PCR conditions and barcode sequences are kept constant.

The differential binding properties of different primers, and secondary structure within target sequences are well-established causes of PCR biases. Multiplex PCRs, for example, frequently show different efficiencies for different primer/target combinations. This bias is a known confounder of T cell repertoire studies, for example. As a result, we and others^16^ have developed techniques that use various types of 5′ RACE, and thus can amplify with amplicon-independent primers. However, the variation in target sequence to be amplified is obviously a variable that cannot be avoided. In this study we therefore consider the extent to which amplification bias can be attributed to sequence variability. We compare the amplification of heterogeneous mixtures of alpha or beta T cell receptor chains (typically containing >10^4 different sequences) with amplification of a monoclonal T cell receptor from a T cell clone (this clone in fact expresses more than one TCR chain, a common feature of T cells^31^). Unexpectedly, PCR amplification efficiency (measured by the number of observed molecules derived from a single ancestor) varies broadly, both for the polyclonal and monoclonal populations. Indeed the extent of variability is very similar, suggesting that the actual sequence of the TCR variable region is not the major cause of different amplification rates. Our results do not, of course, imply that all sequences will be amplified equally. Indeed the length of the target and the GC content are well known to influence PCR efficiency^6^. Rather our results suggest that even when amplifying relatively small amplicons (<1 KB) whose sequences are all rather comparable, substantial variation remains. Some degree of heterogenous amplification of T and B cell receptors has been observed previously^32^,^33^,^34^, although these studies have not focused on analysis of the distribution of the variation, or its relationship to inherent stochasticity of the PCR process.

Our data suggest that the sequence of the ligated barcodes is not the cause of the observed differential amplification, since barcode family size is not correlated between experiments. Although previous studies have shown a small effect of sequence variability adjacent to the PCR primer on efficiency^3^, we have directly compared placing the random barcode sequences immediately next to the primer, or at a distance of six base pairs, and did not observe any significant difference in heterogeneity. Indeed it seemed a priori unlikely that if the variation cannot be attributed to differences between V region sequences it could be caused by 12 base pair barcodes. Additionally, analysis of the structure of the pool of the random barcodes that are used to label the initial molecules suggests that while there is potential for barcode ‘clashes’ (where the same barcode is chosen to label more than one initial molecule), these are not large enough or prevalent enough to be the reason for the large barcode family sizes observed. We do, however, present some theoretical and simulation results that can help to guide the size of barcode pool size in different scenarios. These results suggest that a barcode of 12 base pairs (providing in the order of 10^7^ different sequences) is sufficient to label pools of DNA targets in the order of 10^6^ molecules.

The bulk conditions in all the PCR reactions obviously cannot account for the intra-experimental variation. However, as discussed previously, PCR is by its nature a stochastic process since at each cycle a molecule will be either replicated or not replicated with some probability p, which will be less than 1 for all reactions in which replication efficiency is not 100%. For example, the PCR efficiencies in our model system (which we have measured using qPCR on plasmid dilutions) are typically in the order of 80–90%. Furthermore, it is possible that there is local heterogeneity in the PCR vessel itself: for example temperature gradients, or heterogeneity introduced by phase shifts at the plastic/liquid or liquid/gas surfaces. We therefore examined the implications of different models in detail using a branching process PCR simulator.

Simulation demonstrated clearly that lower efficiencies, a range of efficiencies, competition and resource limitation can all introduce some variation in the predicted output of the PCR for different molecules. As might be predicted, the extent of variation increases with cycle number, and with low and more variable efficiencies. The goal of minimising the number of cycles, and maximising efficiency does therefore lower overall expected variance of product molecular counts. However, the extent of the variance due to these properties is limited and does not explain our observed results. The only model we considered that was able to produce substantial variance in output comparable to that observed in Protocol A (Fig. 1c,d, top) is an inherited efficiency model, where all molecules produced from an initial molecule retain the same efficiency though all cycles. This result, too, is related to well-known evolutionary theory where significant divergence can only occur when selection operates on the inherited properties of the individual. A clue to the cause of the observed heterogeneity is provided by the observation that it is reduced by omitting the 3′ single stranded ligation step, and instead using a fixed primer in the V region (Fig. 1d, middle and bottom panels). Since the length of the cDNA molecules produced may be variable, due to incomplete reverse transcription, variation in length or composition of the cDNA at the 3′ end may be sufficient to significantly alter PCR amplification efficiency. Thus for TCR or BCR repertoire sequence, both multiplex PCR and RACE protocols have the potential to introduce substantial heterogeneity in amplification efficiency, which will materially affect quantitative features of the observed repertoire, substantially increasing the range of clone sizes observed (Supplementary Fig. 2). Barcoding therefore becomes essential for accurate quantification of transcript number.

The observed heterogeneity can be ascribed to two stochastic processes, one deriving from sampling of the amplified product for sequencing and one deriving from inherent heterogeneity of the PCR reaction. The relative contribution of each can be observed by comparing the CV of a simulation with no PCR heterogeneity (Supplementary Fig. 4b, model 1) with the other models we have explored (Supplementary Fig. 4b, models 3,5 and 6). It is evident that for models that do not incorporate inherited efficiencies (i.e. models 3, 5) most of the variation can be attributed to sampling.

In conclusion, we consider the implications of our findings for the community routinely using PCR for quantitative analysis of RNA or DNA populations. The major lesson is that molecular barcoding provides an essential tool that can mitigate for the effects of PCR heterogeneity. This is especially important for studies whose primary output is the comparative quantification of many diverse nucleotide fragments within a mixture, such as repertoire analysis. In situations where single molecule barcoding is difficult, or not practical, every effort needs to be taken to maximise the efficiency of the PCR reactions and minimise the number of cycles. In the longer term, single molecule amplification-free DNA sequencers, which are currently in development, may remove the requirement for a PCR amplification step altogether. In the meantime, it continues to be important to appreciate the inherent stochasticity of the PCR process, and its possible effects on quantitative aspects of molecular biology.

## Additional Information

**How to cite this article**: Best, K. *et al.* Computational analysis of stochastic heterogeneity in PCR amplification efficiency revealed by single molecule barcoding. *Sci. Rep.* **5**, 14629; doi: 10.1038/srep14629 (2015).

## Supplementary Material

Supplementary Information

## Figures and Tables

**Table 1:** Sequences of primers used. N represents a random (unknown) nucleotide and X represents a variable but known nucleotide.

αRC2	GAGTCTCTCAGCTGGTACACG
βRC2	ACACAGCGACCTCGGGTGGGAA
T4DNA_6N_SP2	[Phos]NNNNNNAGATCGGAAGAGCACACGTCTGAACTCCAGTCAC[SpcC3]
αRC1	ACGGCAGGGTCAGGGTTCTGGATAT
βRC1.1	GGTGGGAACACCTTGTTCAGGTCCTC
βRC1.2	GGTGGGAACACGTTTTTCAGGTCCTC
SP1	ACACTCTTTCCCTACACGACGCTCTTCCGATCT
SP2	GTGACTGGAGTTCAGACGTGTGCTCTTCCGATCT
SP1-6N-I-X-αRC1	ACACTCTTTCCCTACACGACGCTCTTCCGATCTNNNNNNXXXXXXACGGCAGGGTCAGGGTTCTGGATAT
SP1-6N-I-X-βRC1	ACACTCTTTCCCTACACGACGCTCTTCCGATCTNNNNNNXXXXXXGGTGGGAACACC(G)TTG(T)TTCAGGTCCTC
P5-SP1	AATGATACGGCGACCACCGAGATCTACACTCTTTCCCTACACGACGCTCTTCC
P7-LX	CAAGCAGAAGACGGCATACGAGATXXXXXXGTGACTGGAGTTCAGACGTGTGCTCTTCCGATC
P5	AATGATACGGCGACCACCGAGATC
P7	CAAGCAGAAGACGGCATACGAGAT
SP1-IX-12N-βRC1.1	ACACTCTTTCCCTACACGACGCTCTTCCGATCTXXXXXXNNNNTNNNNTNNNGGTGGGAACACCTTGTTCAGGTCCTC
SP1-12N-IX-βRC1.1	ACACTCTTTCCCTACACGACGCTCTTCCGATCTNNNNTNNNNTNNNNXXXXXXGGTGGGAACACCTTGTTCAGGTCCTC
VBKT2_1	CTTGGCTATGTGGTCCTTTGC

## References

[b1] Polz M. F. & Cavanaugh C. M. Bias in template-to-product ratios in multitemplate PCR. Appl. Environ. Microbiol. 64, 3724–3730 (1998). 9758791 10.1128/aem.64.10.3724-3730.1998 PMC106531

[b2] Kurata S. *et al.* Reevaluation and Reduction of a PCR Bias Caused by Reannealing of Templates. Appl. Environ. Microbiol. 70, 7545–7549 (2004). 15574958 10.1128/AEM.70.12.7545-7549.2004 PMC535213

[b3] Pan W. *et al.* DNA polymerase preference determines PCR priming efficiency. BMC Biotechnol. 14, 10 (2014). 24479830 10.1186/1472-6750-14-10 PMC3937175

[b4] Alon S. *et al.* Barcoding bias in high-throughput multiplex sequencing of miRNA. Genome Res. 21, 1506–1511 (2011). 21750102 10.1101/gr.121715.111 PMC3166835

[b5] Day D. Identification of non-amplifying CYP21 genes when using PCR-based diagnosis of 21-hydroxylase deficiency in congenital adrenal hyperplasia (CAH) affected pedigrees. Hum. Mol. Genet. 5, 2039–2048 (1996). 8968761 10.1093/hmg/5.12.2039

[b6] Aird D. *et al.* Analyzing and minimizing PCR amplification bias in Illumina sequencing libraries. Genome Biol. 12, R18 (2011). 10.1186/gb-2011-12-2-r18 PMC3188800 21338519

[b7] Bentley D. R. *et al.* Accurate whole human genome sequencing using reversible terminator chemistry. Nature 456, 53–59 (2008). 18987734 10.1038/nature07517 PMC2581791

[b8] Margulies M. *et al.* Genome sequencing in microfabricated high-density picolitre reactors. Nature 437, 376–80 (2005). 16056220 10.1038/nature03959 PMC1464427

[b9] Weinstein J. A., Jiang N., White R. A., Fisher D. S. & Quake S. R. High-Throughput Sequencing of the Zebrafish Antibody Repertoire. Science (80-.). 324, 807–810 (2009). 10.1126/science.1170020 PMC3086368 19423829

[b10] Nagalakshmi U. *et al.* The Transcriptional Landscape of the Yeast Genome Defined by RNA Sequencing. Science (80-.). 320, 1344–1349 (2008). 10.1126/science.1158441 PMC2951732 18451266

[b11] Miner B. E. Molecular barcodes detect redundancy and contamination in hairpin-bisulfite PCR. Nucleic Acids Res. 32, e135–e135 (2004). 15459281 10.1093/nar/gnh132 PMC521679

[b12] McCloskey M. L., Stöger R., Hansen R. S. & Laird C. D. Encoding PCR Products with Batch-stamps and Barcodes. Biochem. Genet. 45, 761–767 (2007). 17955361 10.1007/s10528-007-9114-x

[b13] Casbon J. A., Osborne R. J., Brenner S. & Lichtenstein C. P. A method for counting PCR template molecules with application to next-generation sequencing. Nucleic Acids Res. 39, e81–e81 (2011). 21490082 10.1093/nar/gkr217 PMC3130290

[b14] Kivioja T. *et al.* Counting absolute numbers of molecules using unique molecular identifiers. Nat. Methods 9, 72–74 (2011). 22101854 10.1038/nmeth.1778

[b15] Mamedov I. Z. *et al.* Preparing Unbiased T-Cell Receptor and Antibody cDNA Libraries for the Deep Next Generation Sequencing Profiling. Front. Immunol. 4, 456 (2013). 24391640 10.3389/fimmu.2013.00456 PMC3870325

[b16] Shugay M. *et al.* Towards error-free profiling of immune repertoires. Nat. Methods 11, 653–5 (2014). 24793455 10.1038/nmeth.2960

[b17] Jacob C. & Peccoud J. Estimation of the parameters of a branching process from migrating binomial observations. Adv. Appl. Probab. 30, 948–967 (1998).

[b18] Lalam N., Jacob C. & Jagers P. Modelling the PCR Amplification Process by a Size-Dependent Branching Process and Estimation of the Efficiency. Adv. Appl. Probab. 36, 602–615 (2004).

[b19] Hanlon B. & Vidyashankar A. N. Inference for Quantitation Parameters in Polymerase Chain Reactions via Branching Processes With Random Effects. J. Am. Stat. Assoc. 106, 525–533 (2011).

[b20] Stolovitzky G. & Cecchi G. Efficiency of DNA replication in the polymerase chain reaction. Proc. Natl. Acad. Sci. USA 93, 12947–12952 (1996). 8917524 10.1073/pnas.93.23.12947 PMC24026

[b21] Whitney S. E., Sudhir A., Nelson R. M. & Viljoen H. J. Principles of rapid polymerase chain reactions: mathematical modeling and experimental verification. Comput. Biol. Chem. 28, 195–209 (2004). 15261150 10.1016/j.compbiolchem.2004.03.001

[b22] Aach J. & Church G. M. Mathematical models of diffusion-constrained polymerase chain reactions: basis of high-throughput nucleic acid assays and simple self-organizing systems. J. Theor. Biol. 228, 31–46 (2004). 15064081 10.1016/j.jtbi.2003.12.003

[b23] Gevertz J. L., Dunn S. M. & Roth C. M. Mathematical model of real-time PCR kinetics. Biotechnol. Bioeng. 92, 346–355 (2005). 16170827 10.1002/bit.20617

[b24] Cobbs G. Stepwise kinetic equilibrium models of quantitative polymerase chain reaction. BMC Bioinformatics 13, 203 (2012). 22897900 10.1186/1471-2105-13-203 PMC3519511

[b25] Pienaar E., Theron M., Nelson M. & Viljoen H. A quantitative model of error accumulation during PCR amplification. Comput. Biol. Chem. 30, 102–111 (2006). 16412692 10.1016/j.compbiolchem.2005.11.002 PMC1544370

[b26] Rubin E. A mathematical model and a computerized simulation of PCR using complex templates. Nucleic Acids Res. 24, 3538–3545 (1996). 8836180 10.1093/nar/24.18.3538 PMC146141

[b27] Demotz S. *et al.* Delineation of several DR-restricted tetanus toxin T cell epitopes. J. Immunol. 142, 394–402 (1989). 2463305

[b28] Thomas N., Heather J., Ndifon W., Shawe-Taylor J. & Chain B. Decombinator: a tool for fast, efficient gene assignment in T-cell receptor sequences using a finite state machine. Bioinformatics 29, 542–550 (2013). 23303508 10.1093/bioinformatics/btt004

[b29] Hall B. *et al.* In Curr. Protoc. Mol. Biol. **Chapter 24,** Unit 24.2 (John Wiley & Sons, Inc., 2009).

[b30] Peccoud J. & Jacob C. Theoretical uncertainty of measurements using quantitative polymerase chain reaction. Biophys. J. 71, 101–108 (1996). 8804593 10.1016/S0006-3495(96)79205-6 PMC1233461

[b31] Dash P. *et al.* Paired analysis of TCRα and TCRβ chains at the single-cell level in mice. J. Clin. Invest. 121, 288–295 (2011). 21135507 10.1172/JCI44752 PMC3007160

[b32] Shiroguchi K., Jia T. Z., Sims P. A. & Xie X. S. Digital RNA sequencing minimizes sequence-dependent bias and amplification noise with optimized single-molecule barcodes. Proc. Natl. Acad. Sci. 109, 1347–1352 (2012). 22232676 10.1073/pnas.1118018109 PMC3268301

[b33] Vollmers C., Sit R. V., Weinstein J. A., Dekker C. L. & Quake S. R. Genetic measurement of memory B-cell recall using antibody repertoire sequencing. Proc. Natl. Acad. Sci. 110, 13463–13468 (2013). 23898164 10.1073/pnas.1312146110 PMC3746854

[b34] Shugay M. *et al.* Towards error-free profiling of immune repertoires. Nat. Methods 11, 653–655 (2014). 24793455 10.1038/nmeth.2960

