# Supplementary material for: Computational analysis of stochastic heterogeneity in PCR amplification efficiency revealed by single molecule barcoding
Source: Sci Rep. 2015 Oct 13;5:14629. doi: 10.1038/srep14629 (PMC4602216; doi:10.1038/srep14629)

Computational analysis of stochastic heterogeneity in PCR amplification efficiency revealed by single molecule barcoding.

Katharine Best, Theres Oakes, James M. Heather, John Shawe-Taylor, Benny Chain

Supplementary Information

Supplementary figure legends

Supplementary Figure 1
The proportion of sequenced KT2 TCR molecules (α chain in (a), β chain in (b)) categorised by Decombinator as each of the identified TCR clonotypes. The largest identified clonotypes are coloured and described by the five-part Decombinator identifier (V region, J region, V deletions, J deletions, junctional nucleotides).

Supplementary Figure 2
Details of the effect of the correction of sequencing error and biased PCR amplification on sequencing runs of TCRs from healthy volunteers for alpha and beta samples at two different time points from 4 healthy volunteers (Figures (i) – (iv)).

Top panels: The number of distinct TCR clones observed in the indicated sequencing run of healthy volunteer PB when barcodes are not considered ('raw') and when barcodes are used to correct for PCR amplification bias and sequencing error ('corrected').

Bottom panels: The correlation between TCR clone size observed in the raw or corrected data of the indicated sequencing run of healthy volunteer PB.

Supplementary Figure 3

(a) Black circles: The proportion of barcodes that are observed with the given number of labelling events across all healthy volunteer PBMC sequencing runs in this study. Coloured lines: fitted mixed Poisson models, zero-truncated to account for unobservable zeros. The distributions considered are the zero-truncated Poisson (ZTP), the zero-truncated mixed Poisson-Gamma (ZTPG), and the zero-truncated mixed Poisson-Lognormal (ZTPLN). Zero-truncation occurs after mixing, and best fitting parameters are found via maximum likelihood estimation.
(b) From the parameters of the best-fitting ZTPLN in (a), the inferred structure of the available barcode pool, giving the distribution of barcode copy numbers in a pool of 10^8 oligos.

Supplementary Figure 4

(a) The coefficient of variation (standard deviation/mean) for each sample sequenced under the polyclonal protocol, the monoclonal protocol A and monoclonal protocol B.

(b)-(d) The coefficient of variation of the output from 50 repeated simulations of (a) PCR model 1, (b) PCR model 3, (c) PCR model 5 and (d) PCR model 6 shown alongside the experimentally observed coefficient of variation. Model parameters represented by colour.

The 95% confidence intervals for the CVs for each model and set of parameters shown are :
Model 1: 0.3: (0.60, 0.63); 0.5: (0.57, 0.59); 0.7: (0.54, 0.56); 0.9: (0.51, 0.53).

Model 3: 10: (0.50,0.53); 100: (0.50, 0.52); 1000: (0.50, 0.52); 10000: (0.50, 0.52).
Model 5: [0.5, 0.1]: (0.57, 0.59); [0.5, 0.25]: (0.56, 0.58); [0.9, 0.1]: (0.51, 0.53); [0.9, 0.25]: (0.52, 0.54)
Model 6: [0.45, 0.2, 2.5]: (2.31, 2.49); [0.7, 0.1, 2.5]: (1.36, 1.49); [0.8, 0.2, 2.5]: (0.76, 0.79); [0.95, 0.1, 1]: (0.56, 0.58); [0.95, 0.1, 2.5]: (0.62, 0.64).

Supplementary Figure 5

Observed barcode family size distributions observed under different models of PCR duplication. Simulations performed with 10,000 initial molecules, 25 cycles of PCR (with no error) and sequencing of 10,000 molecules selected from the amplified pool. Simulations were repeated 10 times and the mean and standard deviation are shown. The dotted lines represent the expected distribution if every initial molecule is labelled uniquely and represented equally in the amplified pool.

(a) Model 2: PCR cycles with target degradation. In each cycle, a molecule replicates with probability 0.8. If successful replication does not occur, degradation of that target molecule occurs with the indicated probability.
(b) Model 4: Resource degradation model of PCR. An initial amount of abstract ‘resource’ is available at the start of the process, given as a multiple (the ‘initial resource multiple’) of the number of initial molecules. The efficiency of the reaction in a given cycle depends on the amount of resource available. A successful replication depletes resource at a fixed rate, here 0.5.

Supplementary Figure 6

Schematic of Protocol A (using single strand ligation)

A: TCR RNA is reverse transcribed using oligonucleotides directed against the 5’ of the alpha and beta constant regions (blue arrow). Red circle represents mRNA 5’ cap.

B: RACE is achieved through ligation of DNA adapter to the 3’ end of the cDNA; adapter consists of Illumina sequencing primer SP2 and a hexamer of random nucleotides (6N).

C: Separate single round second (i) and third strand (ii) reactions allow incorporation of another random hexamer at the other end of the amplicons (as well as SP1, the other sequencing primer, and an index for demultiplexing), completing the unique 12-mer barcoding of TCR cDNA.

D: A four-cycle PCR is used to add: another index (that which is sequenced in the dedicated Illumina indexing read) and P7 at one end (i) and P5 at the other (ii). P5 and P7 are the elements required for cluster generation, as they bind to the oligonucleotides that coat the flow-cell, permitting bridge amplification.

E: A final PCR directed against the P5 and P7 elements (for 23 cycles) amplifies full-length amplicons to sufficient concentrations for sequencing. Amplicons are finally purified, quantified, sized and normalised before sequencing on the MiSeq.

Empirical distribution of barcodes

Aggregating barcode data from a number of sequence runs allows us to infer information about the structure of the pool of barcode-oligos. We counted barcode labelling events across our experiments (Supplementary Fig 2a, black dots) and found that the majority of barcodes we observed have only been seen in one labelling event while some have been observed up to 12 times. If the pool of barcodes was uniformly distributed we would expect the barcode labelling event counts to be zero-truncated Poisson distribution, while if the pool follows some other distribution we expect the barcode labelling event counts to follow

a zero-truncated mixed Poisson distribution. We fitted, via maximum likelihood, zero-truncated Poisson and mixed Poisson distributions, and saw that the zero-truncated Poisson mixed with a lognormal distribution provided the best fit to the observed barcode labelling event counts (Supplementary Fig 2a, coloured lines). This suggests that the pool of barcodes we are labelling our molecules from is lognormally distributed.

From the fitted parameters of the zero-truncated Poisson-lognormal distribution we are able to infer the structure of the available barcode pool (Supplementary Fig 2b), showing that in a pool of 10^8 barcode-oligos most labels occur fewer than 5 times while a small proportion occur up to 30 times.

Zero-truncated mixed Poisson model functions

Zero-truncated mixed Poisson models are fitted to data via maximum likelihood optimisation in R of the following functions. Maximum likelihood optimisation was performed using the maxNR of the MaxLik package [1].

```
p_ztp <- function(param, Y) {
  lambda <- param
  (exp(-lambda)*lambda^Y)/(factorial(Y)*(1-exp(-lambda)))
}

ll_ztp <- function(param, Y, K) {
  sum(K * log(p_ztp(param, Y)))
}

p_ztpln <- function(param, Y) {
  mu <- param[1]
  sigma <- param[2]
  n <- length(Y)
  out <- rep(NA, n)
  for (i in 1:n) {
    outside <- 1/(sqrt(2*pi)*factorial(Y[i])*sigma)
    inside <- function(lambda) {
      lambda^(Y[i]-1)*exp(-lambda-(log(lambda)-mu)^2/(2*sigma^2))
    }
    int <- integrate(inside, 0, Inf)
    zt_outside <- 1/(sqrt(2*pi)*sigma)
    zt_inside <- function(lambda) {
      lambda^(-1)*exp(-lambda-(log(lambda)-mu)^2/(2*sigma^2))
    }
    zt_int <- integrate(zt_inside, 0, Inf)
    out[i] <- outside*int$value*(1/(1-zt_outside*zt_int$value))
  }
  return(out)
}

ll_ztpln <- function(param, Y, K) {
  tryCatch( sum(K * log(p_ztpln(param, Y)))
},
error = function(err) {  return(-Inf)  }
  )
}

p_ztpw <- function(param, Y) {
  l <- param[1]
  k <- param[2]
  n <- length(Y)
  out <- rep(NA, n)
  for (i in 1:n){
    outside <- k/(factorial(Y[i]) * l^k)
    inside <- function(lambda) {
      lambda^(Y[i]+k-1)*exp(-lambda-(lambda/l)^k)
    }
    int <- integrate(inside, 0, Inf)
    zt_outside <- k/(l^k)
    zt_inside <- function(lambda) {
      lambda^(k-1)*exp(-lambda-(lambda/l)^k)
```

```
    }
    zt_int <- integrate(zt_inside, 0, Inf)
    out[i] <- outside*int$value*(1/(1-zt_outside*zt_int$value))
  }
  return(out)
}

ll_ztpw <- function(param, Y, K) {
  tryCatch(
{ sum(K * log(p_ztpw(param, Y)))
},
error = function(err) {  return(-Inf)  }
  )
}

p_ztpg <- function(param, Y) {
  mu <- exp(param[1])
  theta <- exp(param[2])
  (   (gamma(Y+theta)/(gamma(theta)*factorial(Y)))   *   (mu^Y   *   theta^theta   /
((mu+theta)^(Y+theta))) * (1/(1-(theta^theta)/(mu+theta)^theta))  )
}

ll_ztpg <- function(param, Y, K) {
  tryCatch(
{ sum(K * log(p_ztpg(param, Y)))
},
error = function(err) {  return(-Inf)  }
  )
}

ll_ztpg <- function(param, Y, X){
  eta <- c(X%*%param[1:(length(param)-1)])
  mu <- exp(eta)
  theta <- exp(param[length(param)])
  sum(   lgamma(Y+theta)   +Y*log(mu)   +   theta*log(theta)   -   lgamma(theta)   -
(Y+theta)*log(mu+theta) - log(1-(theta^theta)/((mu+theta)^theta))  )
}

gr_ztpg <- function(param, Y, X){
  eta <- c(X%*%param[1:(length(param)-1)])
  mu <- exp(eta)
  theta <- exp(param[length(param)])

  db   <-    colSums(X    *    mu    *    (    Y/mu    -    (Y+theta)/(mu+theta)    -
theta^(theta+1)/((mu+theta)^(theta+1) - theta^theta * (mu+theta)) )  )
  dd  <-  theta  *  sum(  digamma(Y+theta)  -  digamma(theta)  +  1  +  log(theta)  -
(Y+theta)/(mu+theta) - log(mu+theta) - theta^theta * (theta/(mu+theta) + log(mu+theta)
- 1 - log(theta))/((mu+theta)^theta - theta^theta)      )

  c(db,dd)
}

hs_ztpg <- function(param, Y, X) {
  eta <- c(X%*%param[1:(length(param)-1)])
  mu <- exp(eta)
  theta <- exp(param[length(param)])

  w   <-   mu*   (      -(theta+Y)/(theta+mu)   +   mu*(theta+Y)/(theta+mu)^2   -
(theta^(theta+1))/((theta+mu)^(theta+1)-theta^theta*(theta+mu)) + mu*theta^(theta+1)*(
(theta+1)*(theta+mu)^theta    -    theta^theta        )/(    (theta+mu)^(theta+1)    -
theta^theta*(theta+mu)  )^2   )

  dbdb <- as.matrix(t(X)%*%Diagonal(length(w), w)%*%X)

  dbdd  <-  colSums(  X  *  mu  *  theta  *  (  (Y+theta)/(theta+mu)^2  -  1/(mu+theta)  +
(theta^(theta+1)*((mu+theta)^(theta-1))/((mu+theta)^theta-
theta^theta)^2)*(theta/(mu+theta)     +log(mu+theta)     -1     -     log(theta))     -
((theta^theta)/((mu+theta)^theta  -  theta^theta))*(1/(mu+theta)  -  theta/(mu+theta)^2)
) )

  A <- digamma(Y+theta) + 1 + log(theta) - digamma(theta) - (Y+theta)/(mu+theta) -
log(mu+theta)  -  ((theta^theta)/((mu+theta)^theta  -  theta^theta))*(theta/(mu+theta)  +
log(mu+theta) -1 - log(theta))
  B   <-   trigamma(Y+theta)   +   1/theta   -   trigamma(theta)   -   2/(mu+theta)   +
(Y+theta)/(mu+theta)^2       -       (-theta/(mu+theta)^2       +       2/(mu+theta)-
1/theta)*(theta^theta)/((mu+theta)^theta   -   theta^theta)   -   (theta/(mu+theta)   +
log(mu+theta) - 1 - log(theta)   )*( theta^theta * (1+log(theta))/((mu+theta)^theta -
```

```
theta^theta) - ((theta^theta)/((mu+theta)^theta - theta^theta)^2)*((mu+theta)^theta *
(theta/(mu+theta)+log(mu+theta)) - theta^theta * (1+log(theta))) )

  dddd <- theta * sum(A) + theta^2 * sum(B)

  h <- rbind(dbdb, dbdd)
  h <- cbind(h, append(dbdd, dddd))
  rownames(h) <- NULL
  h
}
```


1. Henningsen, A. & Toomet, O. maxLik: A package for maximum likelihood estimation in R. *Comput. Stat.* **26,** 443–458 (2010).

Supplementary Figure 1

a

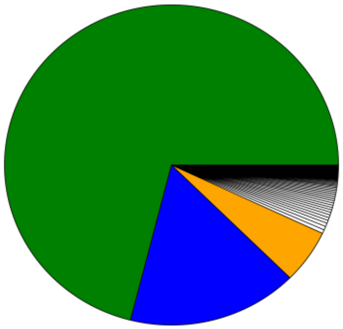

Total KT2 TCRαs sequenced: 6311

Most common TCRs:

Green: 40,3,4,4,CCCG 4484 copies

Blue: 24,48,5,6,TCG 1042 copies

Orange: 19,26,0,5,GACC 342 copies

b

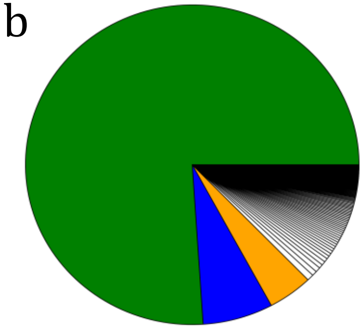

Total KT2 TCRβs sequenced: 6349

Most common TCRs:

Green: 18,1,4,8,CCGGGACAGGTTCGAAA 4828 copies

Blue: 2,7,6,1,CAACGACCTACTTAG 433 copies

Orange: 16,2,10,0,GCCGCGGGGGCGAG 276 copies

Supplementary Figure 2

(i)

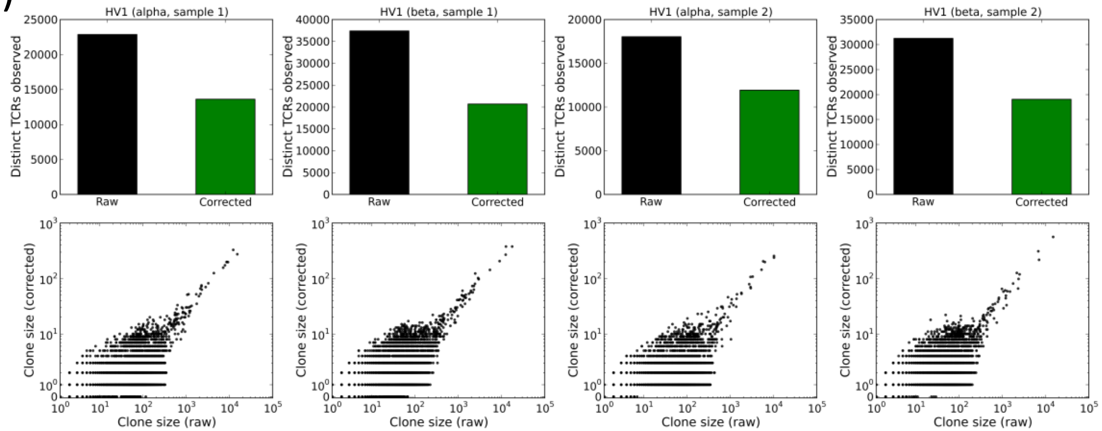

(ii)

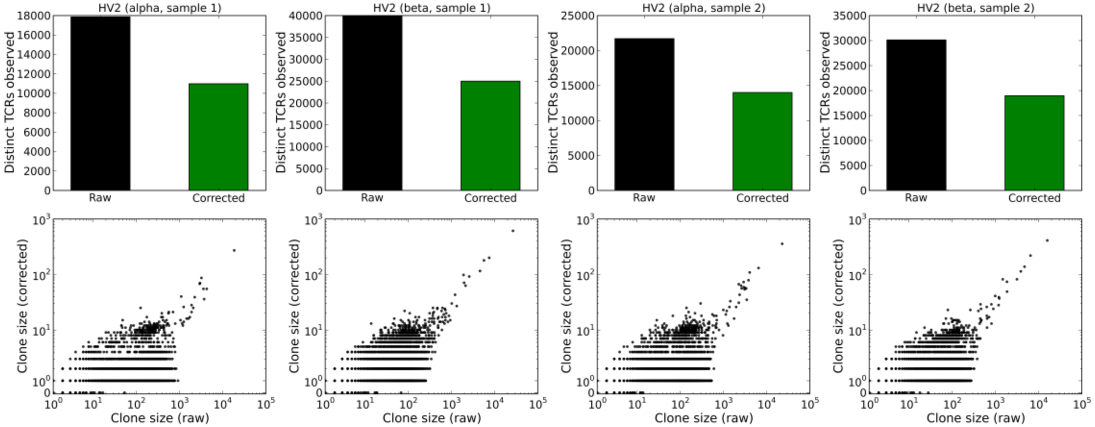

Supplementary Figure 2

(iii)

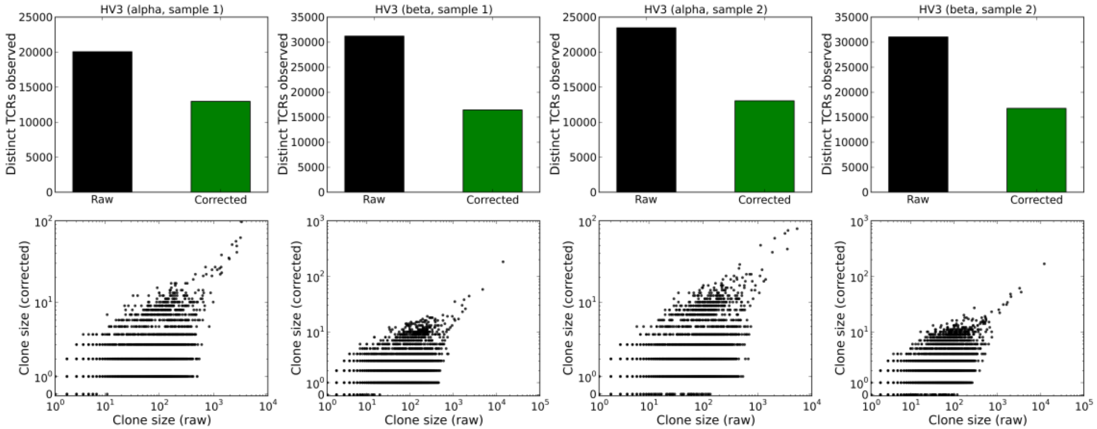

(iv)

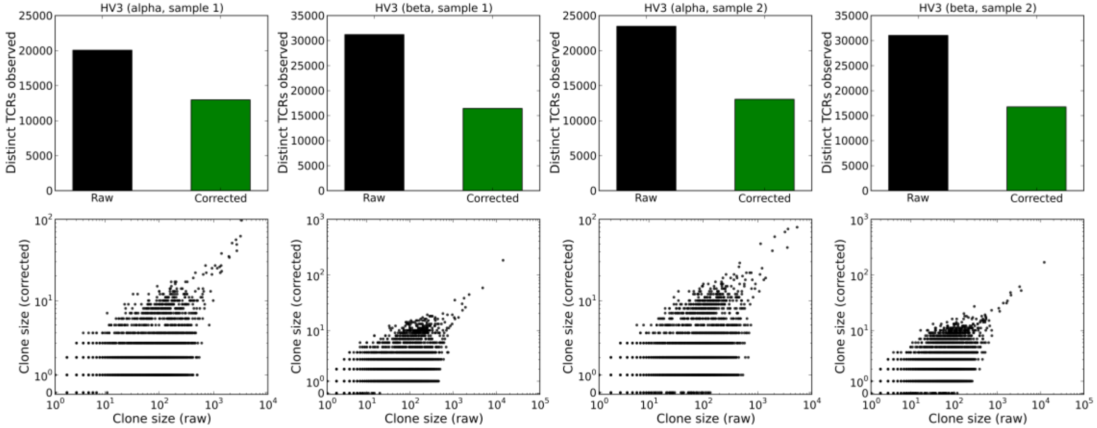

Supplementary Figure 3

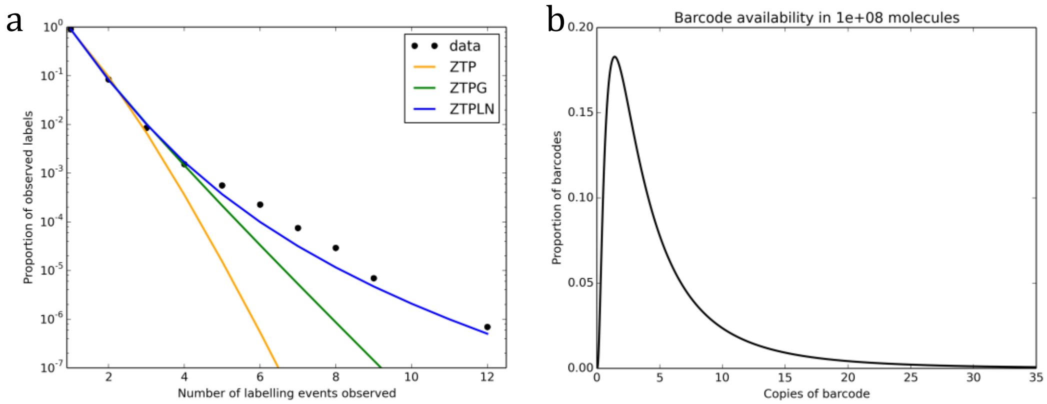

Supplementary Figure 4

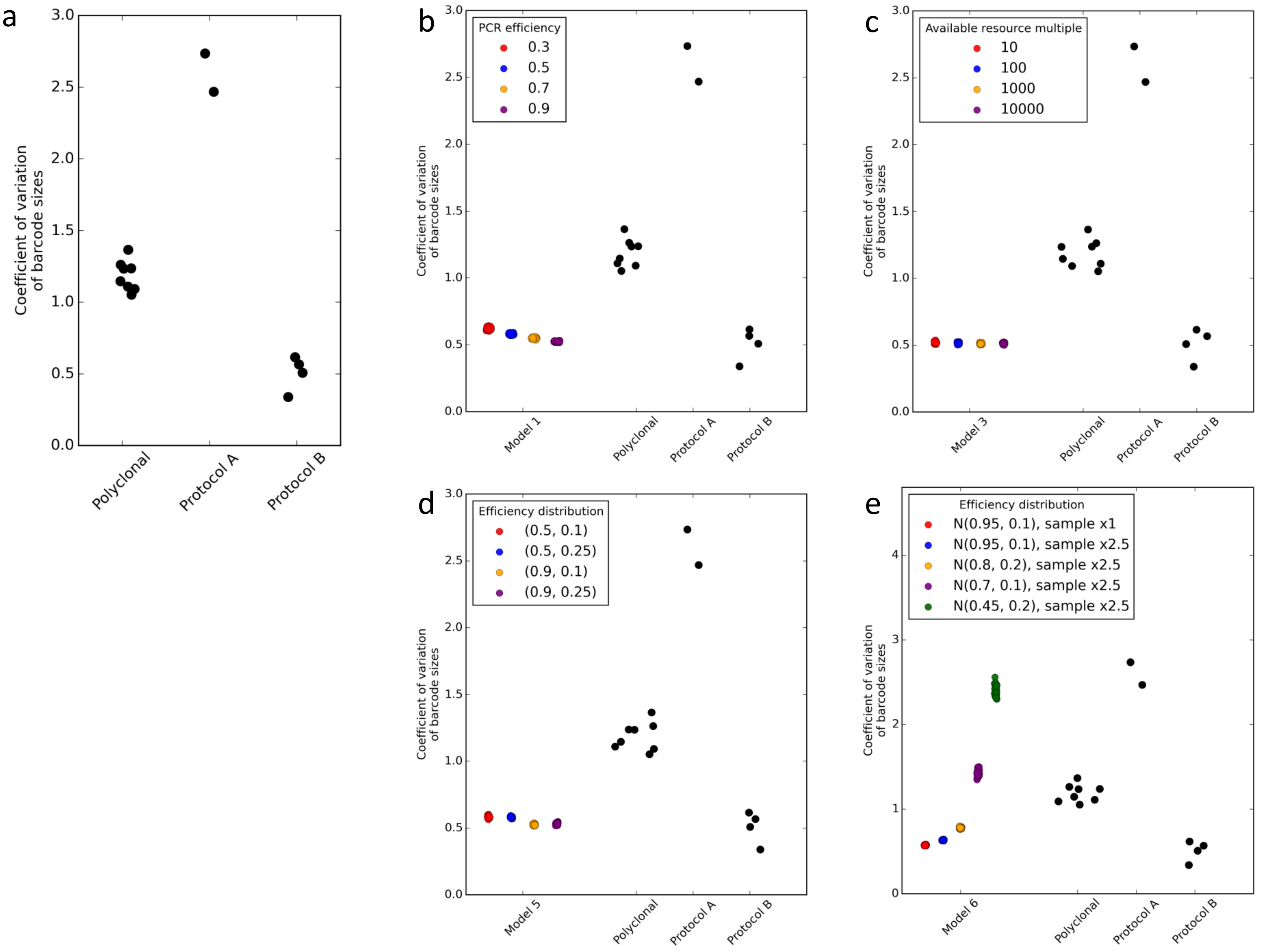

Supplementary Figure 5

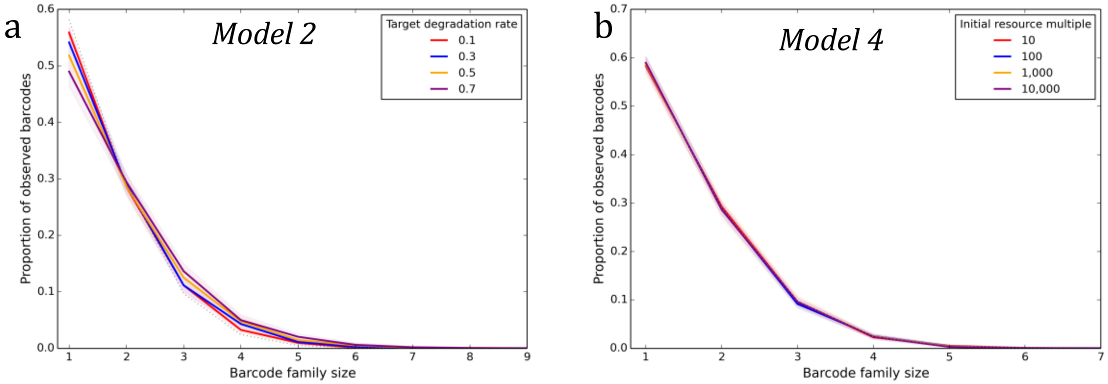

Supplementary Figure 6

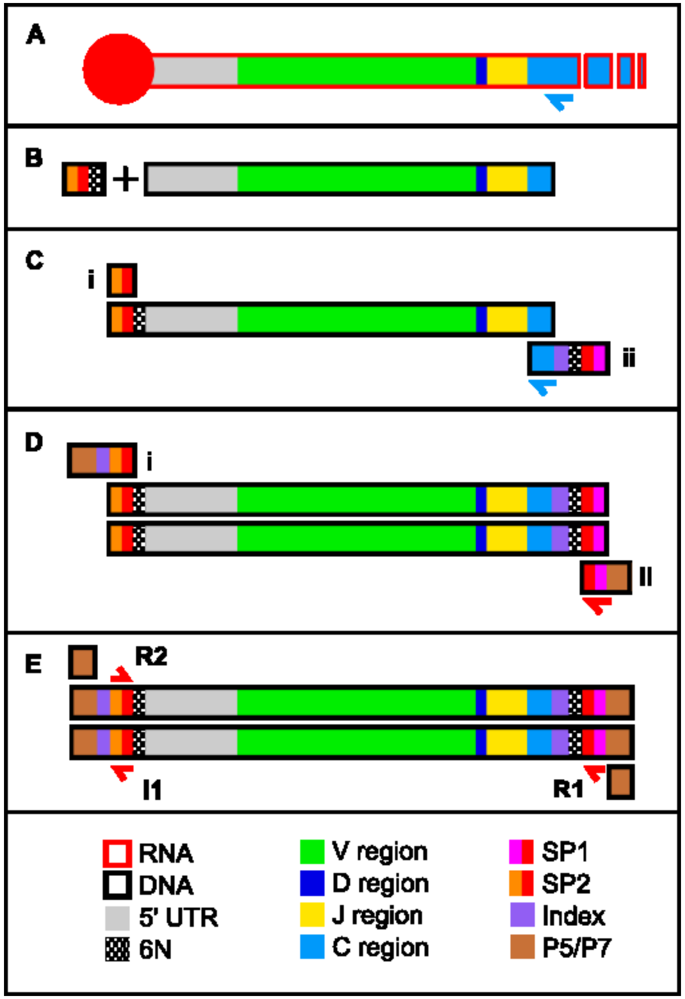

Supplement: Supplementary Information [file srep14629-s1.pdf]
